# Activating values intervention: an integrative pathway to well-being

**DOI:** 10.3389/fpsyg.2024.1375237

**Published:** 2024-04-02

**Authors:** Pninit Russo-Netzer, Ofer Israel Atad

**Affiliations:** ^1^Achva Academic College, Arugot, Israel; ^2^Peres Academic Center, Rehovot, Israel

**Keywords:** well-being, prioritizing meaning, values, positive psychology intervention, life satisfaction

## Abstract

**Introduction:**

The present study expands the existing knowledge base regarding positive psychology interventions (PPIs), by employing an integrative approach to explore the potential benefits of translating values into action.

**Methods:**

Participants (*n* = 476) were randomly assigned to the Activating Values intervention, the affirmation-only, or the control (no treatment) group. The intervention involved participants choosing a life area they valued, affirming its importance, identifying a specific action related to that valued area, and then planning and carrying out that chosen action within the following week. Data was collected at baseline and three follow-up points: one, two, and three weeks after the intervention.

**Results:**

Results suggest that the intervention contributes to the participants’ well-being, including increased self-insight, sense of coherence, and prioritizing meaning, and decreased symptoms of psychopathology. Exploratory content analyses provide a deeper understanding of the content and frequency of activities chosen and the enabling conditions.

**Discussion:**

The discussion explores the findings within this intersection as well as ramifications for brief, scalable interventions to support and promote well-being.

## 1 Introduction

Despite economic prosperity in past decades, individuals today are increasingly experiencing higher levels of mental distress, depression, and anxiety worldwide (e.g., Eisenberg et al., 2007; Twenge et al., 2019). The outbreak of Covid-19 pandemic has intensified mental health challenges such as avoidance, substance abuse, anxiety, stress, depression, and suicidal ideation triggered by the pandemic (e.g., Czeisler et al., 2020; World Health Organization, 2022). The abundance of empirical and practical knowledge regarding human flourishing and the widespread emphasis on happiness and self-fulfillment prevalent in our times have paradoxically yielded undesired outcomes (e.g., Mauss et al., 2011; Ford et al., 2014). This may be due to placing excessive value on and setting unrealistically high standards for happiness, which may make individuals fear not measuring up to expectations (Ehrenreich, 2010; Ford and Mauss, 2014). It also may be effected by the tension between individuals’ efforts and desire to initiate change and their inclination to maintain their current situation, which provides presumed stability. Indeed, a large body of research indicates that when making decisions, people often opt to preserve the existing state of affairs rather than to pursue new actions (e.g., Samuelson and Zeckhauser, 1988; Beshears and Kosowsky, 2020).

Maintaining the default typically demands less cognitive effort and poses less psychological peril than instituting change (Eidelman and Crandall, 2009). This bias toward the status quo stems from the way the human mind processes potential costs and benefits. Specifically, the perceived costs of a change tend to carry more psychological weight than the anticipated gains, even when the benefits objectively outweigh the risks (Moshinsky and Bar-Hillel, 2010). Because the dangers of change loom larger in our mental calculations, the allure of stability and familiarity exerts a strong pull, often leading us to prefer the current situation over unknown alternatives that could improve our circumstances. Thus, enacting change requires cognitive and behavioral effort, overcoming an “activation barrier” (Lewin, 1943), as well as expansion of one’s comfort zone (Russo-Netzer and Cohen, 2022).

The attitude one adopts toward a behavior stands out as a crucial factor in shaping the intention to engage in that behavior or the readiness to change it (Honka et al., 2019). Values, thus, function as essential guiding principles in life, shaping individuals’ perceptions of significance and influencing their decision-making and behavior (Sagiv et al., 2017). As reflections of what individuals perceive as important and worth pursuing in life (Schwartz et al., 2012), values inherently mirror individuals’ motives, needs, and preferences, significantly influencing their daily lives, as well as major life decisions, ultimately shaping their life trajectories and long-term subjective well-being (Schwartz and Sortheix, 2018; Lee and Kawachi, 2019). An individual’s well-being can be affected either by the values they prioritize or by how successfully they fulfill the values that are important to them (Sagiv and Schwartz, 2000).

Successful adoption and maintenance of new patterns of thought and behavior rely heavily on their alignment with one’s inner values and interests. For example, when initiating a behavioral change, intrinsically motivated actions that align with one’s authentic sense of self and fulfill these needs are more likely to be internalized (Ryan and Deci, 2000). Intrinsic and self-concordant goals boost enthusiasm, perceived competence, and long-term persistence (Sheldon and Elliot, 1999). Relatedly, the transtheoretical model of behavioral change holds that for a new behavior to be adopted and sustained, it must be aligned with the individual’s personal values and sense of identity (Prochaska et al., 2008).

Furthermore, research on self-affirmation theory indicates that people are more receptive to difficult or threatening information when they have recently affirmed an important personal value (Sherman and Cohen, 2006). Affirming one’s core values is believed to bolster self-integrity and reduce defensiveness. For example, randomized experiments have shown that brief activities, such as reflecting on one’s core values, can lessen the destructive effects of stress on performance and improve achievement (Cohen and Garcia, 2008; Garcia and Cohen, 2013; Walton and Wilson, 2018). Affirmation protects people from mundane stressors that would otherwise deplete mental resources needed for self-regulation and effective coping by reminding them of what is truly important (Koole et al., 1999; Creswell et al., 2005). However, given that individuals experience greater well-being when they align their actions with their values, the discrepancy between the importance individuals place on their values and their actual behavior poses a challenge (Sheldon and Krieger, 2014; Tessier et al., 2021). This gap suggests that individuals could potentially benefit from psychological support in actively pursuing goals related to their values (Bojanowska et al., 2022).

### 1.1 The present study

A substantial body of evidence suggests that positive psychology interventions (PPIs), defined as deliberate endeavors aimed at augmenting individuals’ overall well-being (Seligman et al., 2005), can initiate lasting enhancements in life satisfaction and potentially alleviating depressive symptoms (Lyubomirsky et al., 2005; Lyubomirsky and Layous, 2013; Margolis and Lyubomirsky, 2020; Ko et al., 2021). These interventions frequently assist individuals in overcoming the phenomenon known as “hedonic adaptation” (Frederick and Loewenstein, 1999), which involves the tendency to become accustomed to both positive and negative life circumstances and experiences (Sheldon and Lyubomirsky, 2004, 2006; Lyubomirsky et al., 2005; Lyubomirsky, 2011). Meta-analyses of PPIs suggest that these interventions hold promise across various contexts, providing accessible and stigma-free preventative measures (Sin and Lyubomirsky, 2009), with an extensive evidence base supporting their effectiveness (Bolier et al., 2013; Carr et al., 2021; Koydemir et al., 2021).

The present study contributes to existing knowledge base regarding PPIs, by taking an integrative approach to investigate how translating values into action may offer potential benefits. Particularly, it builds on previous knowledge regarding values affirmation (Cohen and Garcia, 2008; Garcia and Cohen, 2013) and the prioritizing of such values in everyday life as contributing to desirable outcomes such as well-being (Russo-Netzer, 2019; Russo-Netzer and Shoshani, 2020; Atad and Russo-Netzer, 2022) and takes it a step further by employing an integrative approach to explore potential benefits of translating values into action. More specifically, focusing on one’s values along with execution of an activity that reflects such values were assumed to lead to an improvement of one’s well-being outcomes, such as increased sense of coherence, prioritizing meaning, and self-insight and decreased symptoms of psychopathology. This approach is in line with previous calls, suggesting that “The majority of research undertaken on PPIs has focused primarily on increased positive affect and satisfaction with life and decreased negative affect and depression, as the target outcome variables. Less focus on short-term, hedonic outcomes, to instead include a wider range of well-being outcomes related to eudaimonia, is desirable […] This will align more closely with contemporary conceptualizations of well-being and help to refine the specific well-being outcomes likely to be attained by various PPIs” (Vella-Brodrick, 2012; pp. 348–349).

*Sense of coherence* (SOC) refers to a psychological construct introduced by Antonovsky (1979, 1987) to describe a “global orientation that expresses the extent to which one has a pervasive, enduring though dynamic feeling of confidence that one’s internal and external environments are predictable and that there is a high probability that things will work out as well as can reasonably be expected” (Antonovsky, 1979, p. 123). It consists of three interrelated components: comprehensibility (the belief that situations are understandable), manageability (feeling equipped to cope with life’s challenges and demands), and meaningfulness (feeling that life makes sense and challenges are worthwhile) (Eriksson and Lindström, 2005). Extensive research shows SOC correlates with perceived health, in particular mental health, by enabling effective coping, resilience, and cognitive adaptation to stressors (e.g., Eriksson and Lindström, 2006; Nielsen and Hansson, 2007). We posit that acting in accordance with one’s values can enhance a sense of comprehensibility, manageability, and meaningfulness. This is supported by previous assertions suggesting that maintaining a consistent set of values plays a crucial role in shaping individuals’ perspectives on life and cultivating Sense of Coherence (Sagy and Antonovsky, 2000; Eriksson and Lindström, 2006).

*Prioritizing meaning* refers to the extent to which an individual values and intentionally seeks meaning in life through their goals, behaviors, and activities (Russo-Netzer, 2019). Research suggests that prioritizing meaning is associated with a greater sense of meaning in life, which in turn is positively related to enhanced well-being, such as higher levels of life satisfaction, positive affect, happiness, and gratitude and lower levels of depression and negative affect (e.g., Russo-Netzer, 2019; Russo-Netzer and Bergman, 2019; Russo-Netzer and Shoshani, 2020). In essence, it reflects an active planning and decision-making that weave meaningful activities and situations into daily life routines, self-awareness of one’s core values, and aligning daily activities accordingly. Additionally, it involves fine-tuning these choices by being attuned to any potential shifts in meaning, in a way that is personally meaningful and grounded in everyday experiences.

*Self-insight* refers to the ability to accurately evaluate one’s own personality, emotions, behaviors, and motives (Silvia and Phillips, 2013). It is a key component of self-awareness and metacognition. As defined by Grant (2001), self-insight involves both intrapsychic insight (i.e., understanding one’s own inner experiences) and interpersonal insight (i.e., awareness of how one’s behaviors impact others). High self-insight requires overcoming cognitive biases and integrating candid feedback to form a clear model of the self (Vazire and Carlson, 2011). Successful, purposeful progress through the cycle of self-regulation toward a goal is directed by the individual’s self-insight, which impacts their ability to monitor and evaluate their progress (on cognitive, affective, and behavioral levels) and use such feedback to improve their performance (Boyatzis and Howard, 2013). Research suggests that greater self-insight is associated with improved psychological adjustment, progress in therapy, relationship success, career performance through enhanced self-regulation and resilience, and well-being (Back et al., 2009; Bollich et al., 2015; Selwyn and Grant, 2019). However, increased self-knowledge that could result from self-reflection and self-insight can also lead to lowered self-esteem due to awareness of one’s flaws and ruminative thoughts (Wilson and Dunn, 2004; Lyke, 2009). Thus, self-insight has complex implications for functioning and well-being. We posit that acting in accordance with one’s values can enhance self-insight by potentially affirming and validating their conviction in their values through self-awareness, while building self-confidence.

Overall, we hypothesized that individuals being motivated internally (e.g., Sheldon and Elliot, 1999; Deci and Ryan, 2000) to intentionally choose actions aligned with their core values would benefit their sense of coherence, prioritizing meaning, self-insight, and well-being. The latter was explored by the customary measures of life satisfaction and levels of psychopathology (defined by symptoms of anxiety, depression, and stress).

Our study tested an integrative intervention aimed at facilitating people’s ability to translate values into action. The intervention provided participants with the impetus and opportunity to identify their values and activate them as they chose to in their daily lives. However, given that knowledge about one’s valued life domains and self-awareness are not sufficient to initiate action and may even lead to ruminative thoughts (Harrington and Loffredo, 2010), it was important to understand the nuanced differences between these experiences, beyond a comparison of treatment to nontreatment. Therefore, we conducted a comparison among three groups: an *Activating Values Intervention* (“the intervention”) group (i.e., valued life domains identification and activation through chosen activities), an *affirmation only* group (valued life domains identification with no accompanying action), and a *control-no treatment* group (no valued life domains identification and no action).

Furthermore, in order to deepen and broaden our understanding of the experience of the participants, and because participants self-tailored their activity and self-reported on what they did, we examined, on an exploratory basis, the types of activities they chose to engage in and what contributed to them accomplishing their chosen actions.

## 2 Method

### 2.1 Participants

Participants were recruited via an online panel—a survey platform acknowledged by the Israeli Bureau of Statistics as representing the Israeli population. Recently, online panels have become a common way to target and reach respondents in social science research (e.g., Russo-Netzer and Icekson, 2022). Participants received compensation of $10 for filling in both questionnaires. Ethical approval was obtained from the first author’s academic institution (Approval no. 0182). The study was pre-registered on the Open Science Framework (OSF) before the start of data collection. The registration form can be found on the OSF at: https://osf.io/ju3pf.

The final sample consisted of 476 Israeli adults with a mean age of 32.47 years old (SD = 6.92). Approximately 65% of the sample were women, 43.3% had a BA degree, and 17.2% had an MA degree or higher. As for marital status, 34.9% were single, 61.3% were married, and 3.8% were divorced or widowed.

### 2.2 Measures

We used validated Hebrew versions of established psychological scales used in previous studies.

#### 2.2.1 Subjective well-being

Subjective well-being was measured using the Satisfaction with Life Scale (SWLS; Diener et al., 1985). This scale measures the extent to which people judge their lives to be satisfactory. It includes five items rated on a 7-point Likert scale; the final score is computed as a sum of responses. Items include: “The conditions of my life are excellent” and “I am satisfied with my life” (α = 0.90).

#### 2.2.2 Self-insight

Self-insight was measured using the Self-Reflection and Insight Scale (SRIS-IN; Grant et al., 2002). The SRIS-IN measures individuals’ levels of insight into their thoughts, feelings, and behaviors. It includes eight items rated on a 6-point Likert scale; the final score is computed as a sum of responses. Items include: “I usually know why I feel the way that I do” and “My behaviour often puzzles me” (reverse scored) (α = 0.86).

#### 2.2.3 Psychopathology

Psychopathology was measured using the Depression, Anxiety and Stress Scale (DASS-21; Lovibond and Lovibond, 1995). The DASS-21 comprises three subscales measuring depression, anxiety, and stress, which, when combined, give a total psychopathology score. It includes 21 items rated on a 4-point Likert-type scale (α = 0.94); the final score is computed as a sum of responses.

#### 2.2.4 Sense of Coherence

Sense of Coherence was measured using the Sense of Coherence scale (SOC; Antonovsky, 1987). The SOC measures comprehensibility, manageability and meaningfulness. It includes 29 items rated on a 7-point Likert-type scale (α = 0.92); the final score as well as scale components are computed as a sum of responses.

#### 2.2.5 Prioritizing Meaning

Prioritizing Meaning was measured using the Prioritizing Meaning Scale (Russo-Netzer, 2019). The Prioritizing Meaning Scale measures the extent to which individuals intentionally act and organize, as well as make decisions, in their day-to-day life so that they can experience more meaning. It includes 12 items rated on a 9-point scale (from *1 = disagree strongly* to *9 = agree strongly*) (α =0.95), including: “I prefer to engage in activities which are related to the sense of meaning in my life” and “The manner in which I organize my day reflects values that are meaningful to me.”

#### 2.2.6 Control variables

Several sociopsychological and demographic variables that have been found to be associated with meaning and life satisfaction were controlled for in the statistical analysis. These include age (Vaillant, 2008), gender (Meisenberg and Woodley, 2015), marital status (Boyce et al., 2016), parenting (Umberson et al., 2010; Corbett, 2018), and religiosity (Van Cappellen et al., 2016). Parenting was coded as the participants’ response to the question: “Do you have children?” (*0 = no, 1 = yes*). Religiosity was coded as participants’ response to the question: “In terms of religion, I consider myself…” (*0 = secular, 1 = nonsecular*).

#### 2.2.7 Open-ended responses

Participants in the intervention group were also asked to record their responses to a free-response question about their experience. Specifically, at T2, the participants were asked to describe in their own words the activity they chose to do, what enabled them to perform their chosen activity, and what they learned about themselves following their experience.

### 2.3 Design

Each participant was randomly assigned to one of the following three groups: Group 1 (the intervention, including both affirmation and action), Group 2 (affirmation only), or Group 3 (control-no treatment). Participants were asked to complete an online questionnaire at four points: (1) After randomization (T_1_); (2) one week later (T_2_); (3) two weeks later (T_3_); and (4) three weeks later (T_4_).

### 2.4 Procedure

**Figure 1:**
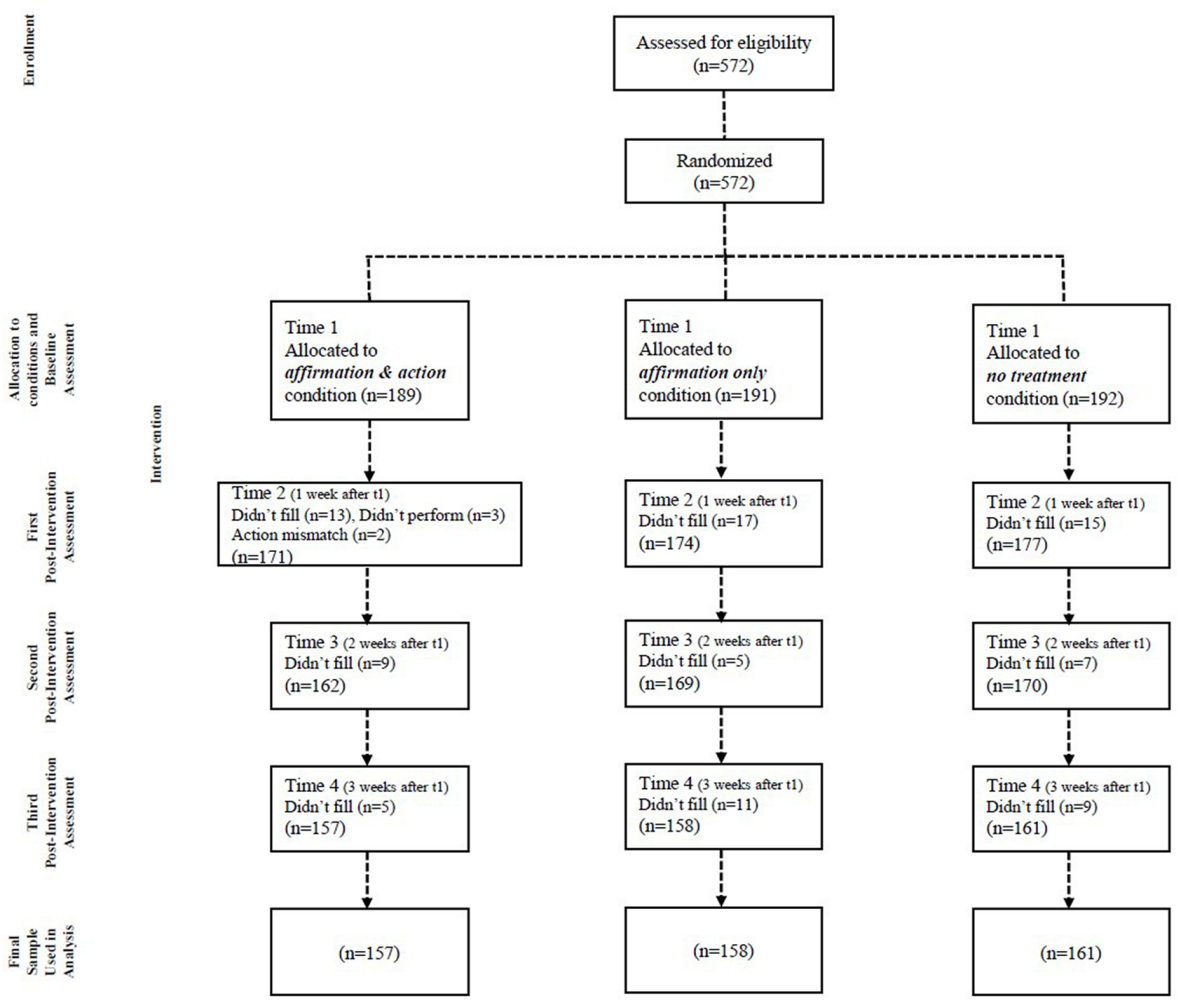
Flow of participants through each stage of the study.

Figure 1 presents the flow of participants through the three arms of the study. At baseline (T1), 572 participants were randomly assigned to one of the three groups and completed the pretest questionaries. At T2 (assessment one week later), participants were asked to fill out their assigned questionnaires. Of the 572 who completed baseline questionaries, 476 (83%) completed all follow-up assessments at T2, T3 and T4 and their assigned intervention. We excluded 91 participants who did not fill out questionnaires (45 participants at T2, 21 participants at T3, and 25 participants at T4). Participants who completed only part of the questionnaires did not differ significantly from those who completed all questionnaires, in terms of their responses to the questionnaire at T1 and their demographic characteristics. There was no differential attrition by condition. Of the 189 participants in the intervention group (affirmation and action) present at baseline (T1), a total of 157 filled out all their assigned questionnaires at T2, T3 and T4 (83%). Of the 191 participants in the affirmation only group present at baseline (T1), a total of 158 filled out all their assigned questionnaires at T2, T3 and T4 (83%). Of the 192 participants in the control-no treatment group present at baseline (T1), a total of 161 filled out all their assigned questionnaires at T2, T3 and T4 (84%). In addition, in the intervention group, the action selected at T1 was compared to the action reported as performed at T2. If a mismatch was found (*n* = 2), or if the action selected was not performed (*n* = 3), the participant was excluded from the analysis. The final sample (*n* = 476) exceeds Cohen’s (2013) recommendation (of 140) for achieving a power of 0.85.

#### 2.4.1 Intervention group

At T1, participants in this group filled out a questionnaire containing the scale items listed in the Measures section. In the final question, participants were asked to (1) examine the main areas of their lives (e.g., work, health, nutrition, sleep, fun and recreation, friends, family, career, personal development, physical activity, work-life balance, spiritual development, or significant other) and choose a life area they viewed as valuable to them; (2) affirm the importance of this life area in their lives by reflecting on what makes this domain meaningful and significant for them, as well as considering how their life would be like if a change will occur in it; (3) identify a specific action they could perform in relation to this life area within the following week; (4) plan and schedule the selected action; and (5) perform the chosen action within the following week. At T2, participants declared whether they completed their chosen action and reported on the activity they performed in the previous week. Then, they re-filled out the questionnaire again, with the open-ended question replacing the initial final question. At T3 and T4, the participants filled out again the questionnaire with the same scale items listed in the Measures section.

#### 2.4.2 Affirmation only group

At T1, participants in this group filled out a questionnaire containing the scale items listed in the Measures section. The final question in this group changed to ask the participants to (1) examine the main areas of their lives (e.g., work, health, nutrition, sleep, fun and recreation, friends, family, career, personal development, physical activity, work-life balance, spiritual development or significant other) and choose a life area they viewed as valuable to them and that they wished to focus on; and (2) affirm the importance of this life area in their lives.

At T2, T3 and T4, the participants filled out again the questionnaire with the same scale items listed in the Measures section.

#### 2.4.3 Control-no treatment group

At T1, T2, T3 and T4, participants in this group filled out the questionnaire that included the scale items listed in the Measures section and the measures listed in the Control Variables section. The questionnaire at T1 did not include the final question included for the other groups.

### 2.5 Data analysis

#### 2.5.1 Quantitative data analysis

Changes within each group from T1 to T2, T3 and T4 were analyzed using paired-samples *t*-tests. Means, standard deviations, significant levels, and Cohen’s *d* for all groups are shown in Tables 1–3. Means, Standard deviations, significant levels, and Cohen’s *d* from Time 1 to Time 4 for the intervention group are presented in Table 1. Differences between the groups were analyzed using repeated-measures ANOVA followed by planned contrasts. Partial eta squared ( $\eta_{p}^{2})$ and Cohen’s *d* were calculated to demonstrate effect size. Partial eta squared is commonly used to measure the effect size of different variables in ANOVA models. Cohen’s *d* is considered an appropriate indication of effect size for the comparison between two means. Cohen (2013) suggested that *d* = 0.2 be considered a “small” effect size, 0.5 represents a “medium” effect size, and 0.8 and higher a “large” effect size. Before the analyses were conducted, the data were checked for violations of normality. No violations were detected. There were no statistically significant differences between the groups on any measure at T1. In addition, in the intervention group, no statistically significant differences were found on any measure at T1, between those who performed or did not perform their chosen activity or between the reported and performed action (match or mismatch).

**Table 1:** Means, standard deviations, significant levels, and Cohen’s *d* from Time 1 to Time 4 for the intervention group (*n* = 157).

	Time 1		Time 2				Time 3				Time 4			
Variables	M	SD	M	SD	*p*	*d*	M	SD	*p*	*d*	M	SD	*p*	*d*
SWB	27.04	4.45	28.12	3.84	**<0.001**	0.39	27.49	4.01	**0.015**	0.16	27.13	4.17	0.329	0.03
Self-Insight	34.73	6.30	35.75	6.29	**<0.001**	0.24	35.27	6.27	**0.048**	0.13	35.03	6.26	0.176	0.07
Prioritizing Meaning	79.91	16.46	81.51	17.14	**0.047**	0.13	80.98	16.58	0.134	0.08	80.17	16.52	0.396	0.02
SOC total	142.03	20.51	145.97	22.97	**<0.001**	0.37	143.33	21.76	**0.045**	0.13	142.76	20.55	0.168	0.07
Manageability	48.66	8.13	49.99	8.48	**<0.001**	0.26	49.54	8.43	**0.011**	0.17	49.18	8.37	0.091	0.10
Comprehensibility	48.43	10.17	50.97	10.50	**<0.001**	0.48	49.05	10.52	0.057	0.12	48.71	10.19	0.239	0.05
Meaningfulness	44.93	6.04	45.00	6.64	0.410	0.17	44.73	6.39	0.247	0.05	44.88	6.23	0.425	0.01
Psychopathology	13.02	9.97	10.31	9.34	**<0.001**	0.38	12.10	9.87	**0.042**	0.13	12.62	9.89	0.279	0.04

SWB, subjective well-being; SOC, Sense of Coherence. Bold values are significant.

**Table 2:** Means, standard deviations, significant levels, and Cohen’s *d* from Time 1 to Time 4 for the affirmation only group (*n* = 158).

	Time 1		Time 2				Time 3				Time 4			
Variables	M	SD	M	SD	*p*	*d*	M	SD	*p*	*d*	M	SD	*p*	*d*
SWB	26.32	6.38	26.21	6.54	0.370	0.03	26.27	6.47	0.467	0.01	26.53	6.51	0.346	0.06
Self-insight	34.90	5.85	34.53	6.36	0.145	0.09	34.99	6.41	0.369	0.02	34.82	6.28	0.453	0.02
Prioritizing meaning	78.48	16.82	77.42	18.00	0.140	0.09	77.90	17.62	0.449	0.05	78.82	16.89	0.421	0.03
SOC total	140.29	22.45	139.65	25.56	0.302	0.04	139.95	25.25	0.437	0.02	139.87	24.03	0.419	0.03
Manageability	48.63	8.71	48.21	10.14	0.210	0.07	48.02	10.10	0.368	0.05	47.80	10.01	0.439	0.06
Comprehensibility	47.66	9.57	47.94	10.34	0.302	0.04	48.02	9.95	0.348	0.06	48.29	9.44	0.347	0.02
Meaningfulness	43.99	7.69	43.50	8.90	0.185	0.07	43.61	8.96	0.344	0.06	43.77	8.35	0.409	0.03
Psychopathology	13.10	10.66	13.40	11.16	0.341	0.03	13.35	11.30	0.424	0.03	13.13	10.81	0.492	0.03

SWB, subjective well-being; SOC, Sense of Coherence.

**Table 3:** Means, standard deviations, significant levels, and Cohen’s *d* from Time 1 to Time 4 for the control - no treatment group (*n* = 161).

	Time 1		Time 2				Time 3				Time 4			
Variables	M	SD	M	SD	*p*	*d*	M	SD	*p*	*d*	M	SD	*p*	*d*
SWB	26.03	5.40	26.41	5.72	0.215	0.06	26.36	4.58	0.258	0.06	25.97	5.15	0.455	0.09
Self-insight	35.40	6.91	35.03	6.89	0.165	0.08	34.98	6.61	0.125	0.09	35.06	6.59	0.172	0.08
Prioritizing meaning	78.14	17.15	77.68	17.88	0.408	0.02	78.12	17.76	0.496	0.01	78.42	17.56	0.443	0.01
SOC total	139.31	25.03	138.70	24.51	0.343	0.03	139.58	25.47	0.449	0.01	139.93	24.77	0.384	0.02
Manageability	48.14	10.77	47.34	9.74	0.149	0.08	47.94	9.87	0.414	0.02	48.25	10.12	0.452	0.01
Comprehensibility	47.35	12.30	48.13	10.15	0.147	0.08	47.58	10.23	0.381	0.03	48.05	11.53	0.176	0.08
Meaningfulness	43.82	7.01	43.23	8.00	0.119	0.10	44.06	8.04	0.416	0.002	43.63	7.95	0.398	0.01
Psychopathology	13.61	11.95	12.80	12.66	0.135	0.09	13.55	11.25	0.476	0.05	12.89	11.55	0.233	0.06

SWB, subjective well-being; SOC, Sense of Coherence.

#### 2.5.2 Coding of open responses

We undertook, on an exploratory basis, an assessment of the types of activities that participants engaged in, the enabling conditions for executing the activity, and the personal insights gained from their experience. Each participant’s response was evaluated by two trained coders independently, each blind to the hypothesis of the research. The coders sorted the responses into the various content categories, achieving strong inter-rater reliability: Kappa = 0.91 for types of activity, 0.81 for perceived enabling conditions, and 0.84 for insights gained. Before analysis, the assessments of the two coders were reviewed, and any disagreement between them was resolved by an independent third coder, whose “vote” resolved the disagreement between the two. In order to check whether the allocation of participants into different conditions had been conducted in a truly random manner or if any systematic biases had existed, we conducted an Χ ^2^ test, comparing the themes selected by the participants in the intervention group to the ones selected by the participants in the affirmation only group. No significant differences were found, suggesting that the randomization procedure was sufficient.

## 3 Results

### 3.1 Changes within each group

We conducted planned paired *t*-tests between the baseline scores (T1) and the scores on the different follow-ups (T2, T3 and T4).

#### 3.1.1 Subjective well-being

Participants in the intervention group experienced significantly higher levels of SWB at T2 (one week after the intervention) than at T1 (*p* < 0.001, *d* = 0.39). These higher levels of SWB remained significantly elevated at T3 (two weeks after the intervention) (*p* < 0.015, *d* = 0.16), but became non-significant at T4 (three weeks after the intervention) (*p* = 0.329, *d* = 0.03).

#### 3.1.2 Self-insight

Participants in the intervention group experienced significantly higher levels of self-insight at T2 (one week after the intervention) than at T1 (*p* < 0.001, *d* = 0.24). These higher levels of self-insight remained significantly elevated at T3 (two weeks after the intervention) (*p* = 0.048, *d* = 0.13), but became non-significant at T4 (three weeks after the intervention) (*p* = 0.176, *d* = 0.07).

#### 3.1.3 Prioritizing meaning

Participants in the intervention group experienced significantly higher levels of prioritizing meaning at T2 (one week after the intervention) than at T1 (*p* = 0.047, *d* = 0.13). These higher levels of prioritizing meaning became non-significant at T3 (three weeks after the intervention) (*p* = 0.134, *d* = 0.08), and at T4 (*p* = 0.396, *d* = 0.02).

#### 3.1.4 Sense of coherence

Participants in the intervention group experienced significantly higher levels of sense of coherence at T2 (one week after the intervention) than at T1 (*p* < 0.001, *d* = 0.37). These higher levels of sense of coherence remained significantly elevated at T3 (two weeks after the intervention) (*p* = 0.045, *d* = 0.13), but became non-significant at T4 (three weeks after the intervention) (*p* = 0.168, *d* = 0.07).

#### 3.1.5 Psychopathology

Participants in the intervention group experienced significantly lower levels of psychopathology at T2 (one week after the intervention) than at T1 (*p* < 0.001, *d* = 0.38). These lower levels of psychopathology remained significantly decreased at T3 (two weeks after the intervention) (*p* = 0.042, *d* = 0.13), but became non-significant at T4 (three weeks after the intervention) (*p* = 0.279, *d* = 0.04).

Participation in the affirmation only or the control-no treatment group was not associated with significant changes on any measure between T1 and T2, T3 or T4.

### 3.2 Differences between the groups

A three between-subject condition (intervention & affirmation only & control) by four time period repeated measure ANOVA for all dependent variables was conducted. All the controlled variables were treated as covariates.

#### 3.2.1 Subjective well-being

A repeated-measures ANOVA revealed a significant condition-by-time interaction effect for subjective well-being (*F*(2,468) = 3.15, *p* = 0.044, $\eta_{p}^{2}$ = 0.013). To probe the interaction, we conducted planned contrasts comparing the intervention and control conditions at each time point. This analysis revealed that at T2 the change in subjective well-being in the intervention group was significantly greater than the corresponding changes in the affirmation only group (*p* = 0.004; *d* = 0.30) and in the control-no treatment group (*p* = 0.006; *d* = 0.27). These results remained significant at T3 (*p* = 0.013; *d* = 0.27 and *p* = 0.021; *d* = 0.22, respectively), but became non-significant at T4. The affirmation only and the control-no treatment groups did not differ significantly in terms of change in subjective well-being at all points of measurement.

#### 3.2.2 Self-insight

A repeated-measures ANOVA revealed a significant condition-by-time interaction effect for self-insight (*F*(6,1,404) = 5.80, *p* = 0.003, $\eta_{p}^{2}$ = 0.024). To probe the interaction, we conducted planned contrasts comparing the intervention and control conditions at each time point. This analysis revealed that at T2 the change in self-insight in the intervention group was significantly greater than the corresponding changes in the affirmation only group (*p* < 0.001; *d* = 0.37) and in the control-no treatment group (*p* > 0.001; *d* = 0.54). These results remained significant at T3 (*p* = 0.046; *d* = 0.18 and *p* = 0.014; *d* = 0.25, respectively), but became non-significant at T4. The affirmation only and the control-no treatment groups did not differ significantly in terms of change in self-insight at all points of measurement.

#### 3.2.3 Prioritizing meaning

A repeated-measures ANOVA did not find a significant condition-by-time interaction effect for prioritizing meaning (*F*(6,1,404) = 1.03, *p* = 0.358, $\eta_{p}^{2}$ = 0.004).

#### 3.2.4 Sense of coherence

A repeated-measures ANOVA revealed a significant condition-by-time interaction effect for sense of coherence (*F*(6,1,404) = 5.58, *p* = 0.004, $\eta_{p}^{2}$ = 0.023). To probe the interaction, we conducted planned contrasts comparing the intervention and control conditions at each time point. This analysis revealed that at T2 the change in sense of coherence in the intervention group was significantly greater than the corresponding changes in the affirmation only group (*p* < 0.001; *d* = 0.44) and in the control-no treatment group (*p* = 0.003; *d* = 0.31). These results remained significant at T3 (*p* = 0.016; *d* = 0.24 and *p* = 0.020; *d* = 0.23, respectively), but became non-significant at T4. The affirmation only and the control-no treatment groups did not differ significantly in terms of change in sense of coherence at all points of measurement.

Repeated-measures ANOVA for the sense of coherence components revealed significant condition-by-time interaction effects for the manageability and the comprehensibility components (manageability*: F*(6,1,404) = 4.31, *p* = 0.014, $\eta_{p}^{2}$ = 0.018; comprehensibility: *F*(6,1,404) = 4.98, *p* = 0.007, $\eta_{p}^{2}$ = 0.021), but not for the meaningfulness component (*F*(6,1,404) = 0.74, *p* = 0.476, $\eta_{p}^{2}$ = 0.003). To probe the interaction, we conducted planned contrasts comparing the intervention and control conditions at each time point. This analysis revealed that at T2 the change in manageability in the intervention group was significantly greater than the corresponding changes in the affirmation only group (*p* < 0.001; *d* = 0.40) and in the control-no treatment group (*p* = 0.007; *d* = 0.29). These results remained significant at T3 (*p* = 0.028; *d* = 0.21 and *p* = 0.017; *d* = 0.24, respectively), but became non-significant at T4. The affirmation only and the control-no treatment groups did not differ significantly in terms of change in manageability at all point of measurement. The change in comprehensibility in the intervention group was significantly greater at T2 than the corresponding changes in the affirmation only group (*p* < 0.001; *d* = 0.39) and in the control-no treatment group (*p* = 0.015; *d* = 0.24), but became non-significant at T3 and T4. The affirmation only and the control-no treatment groups did not differ significantly in terms of change in comprehensibility at all point of measurement.

#### 3.2.5 Psychopathology

A repeated-measures ANOVA revealed a significant condition-by-time interaction effect for psychopathology (*F*(6,1,404) = 6.10, *p* = 0.002, $\eta_{p}^{2}$ = 0.025). To probe the interaction, we conducted planned contrasts comparing the intervention and control conditions at each time point. This analysis revealed that at T2the change in psychopathology in the intervention group was significantly greater than the corresponding changes in the affirmation only group (*p* < 0.001; *d* = 0.38) and in the control-no treatment group (*p* = 0.017; *d* = 0.24). These results remained significant at T3 (*p* = 0.017; *d* = 0.24 and *p* = 0.034; *d* = 0.20, respectively), but became non-significant at T4. The affirmation only and the control-no treatment groups did not differ significantly in terms of change in psychopathology at all points of measurement.

### 3.3 Analysis of open responses

Table 4 displays the types and frequency of activities for participants in the intervention group. Table 5 includes their responses regarding what enabled them to perform their chosen activities. Table 6 includes their responses as to the personal insights they gained from the experience.

**Table 4:** Content and frequency of activities chosen (Time 1).

Theme	Frequency of theme	Cumulative percentage of theme Frequencies	Experts from participant report
Family relationships	55	34.80	“I made sure to spend time with my daughters all week long – we went to the park, went on a weekend trip – just us for quality time”; “I visited my grandmother despite having an intensive week and not having time”; Family weekend outing and a meal I cooked together with the kids.”
Physical activity	26	51.47	“I completed the number of workouts I set as my goal”; “I did more sports activity than I planned”; “I dedicated to dedicate more time than usual to being active”
Health	23	66.18	“I took actions in the health realm to achieve a blood sugar balance”; I got blood tests done, after long procrastination”; “Health-wise, I went to several doctors for a check-in after a long time not finding the time to do it.”
Romantic relationships	23	80.88	“Deep soulful conversation with my partner”; “Breaking up with my girlfriend, which should have been done long ago; “‘Romantic dinner date.”
Career and finances	11	88.24	“Created a vision for my career and set a goal and steps for next week”; “Reviewed and examined my financial state to take better action”; “Spoke with someone about a new job, sent resumes to several places.”
Hobbies, leisure, reflection	6	92.16	“Spent more time on hobbies to nurture my soul and spiritual aspect”; “Deleted Instagram to spend more time in presence.”
Learning and personal growth	5	95.10	“Listened to a podcast on a topic I’m very interested in”; “Started getting into and learning about an area I previously had no knowledge about.”
Attitude	5	98.04	“Took the time be calmer and focus on positive thoughts”; To communicate with my kids better, not get angry at them all the time.”
Friends	3	100	“Made an effort to be there for a friend going through a hard time”; “Spent time getting together with friends.”
Total	157		

**Table 5:** Enablers to perform the chosen activities (Time 2).

Theme	Frequency of theme	Cumulative percentage of theme frequencies	Experts from participant report
Planning and reminders	30	19.11	“Built a schedule that ‘forced’ me to perform the tasks I need to do according to deadlines”; “Set a reminder on a note about the meeting I planned to attend”; “Allocated defined time during the week.”
Supporting environment	26	35.96	“Consulted with friends who helped me gain clarity and focus”; “Received help from friends for my studies, and the passion and desire to succeed in studies, which pushed me to look for various study methods.”
Will and motivation	26	52.25	“The desire to deepen the relationship with the children and to maintain the partnership.”
Commitment	26	68.55	“It helped me that I committed to perform the action”; “Knowing that I cannot back up now.”
Goal setting and dedication	25	84.47	“I felt that if I set an important goal for myself, I need to stick to it and that I must not fail in the action I defined for myself to perform”; “Determination, the will to achieve the goals I set without giving up on myself.”
Importance of action (value)	12	92.34	“An event important enough to allocate time for it even in the busy period I am currently in”; “Understanding that it is part of what is most important to me in life”; “It means a lot to me so I need to take the effort to achieve it.”
Awareness and significance	12	100	“The understanding that my day will look better and I will feel more satisfaction if I perform the action”; “The knowledge that it fulfills me with satisfaction and drive.”
Total	157		

**Table 6:** Personal insights from the experience (Time 2).

Theme	Frequency of theme	Cumulative percentage of theme frequencies	Experts from participant report
Awareness and personal development	34	21.35	“I learned that it takes time for psychological processes to develop but when they happen in their own time from acceptance, it’s the best”; “I learned that I need to dedicate more time for myself and to the things that matter to me.”
Self-efficacy	28	39.33	“I learned that when I really want something, I have the willpower to do it, I always testify to myself that I do not follow my own wishes, but after this experience I now realize that it’s not true”; “I leaned that if I really want something, I can achieve it.”
Willpower: investment and perseverance	26	56.18	“I learned that in order to achieve what I really want, I need to work hard”; “I realized that if I want something, I need to act for it”; “I learned that anything is possible if there is willpower, if I really want to make it happen”; “I wanted to do this for a long time and finally had the chance because I prioritized it.”
Importance of time investment	22	70.22	“I learned that Instagram consumes my time and now I really have more time to study and to do important things rather than spending my time and energy there”; I learned how important it is to define time in the week for personal goals and stick to it.”
Importance of relationships	22	84.27	“I realized that family is a top value for me, and that I should allocate more time to family relationships”; “I learned that I must continue to nurture the relationship with my loved ones.”
Procrastination and difficulty in achieving goals	17	94.94	“I realized that I cannot achieve the things I set for myself”; “I learned that it’s hard for me to prioritize tasks, I tend to procrastinate.”
Significance leads to action	8	100	“I realized that once a goal is important to me, it’s easier for me to perform it”; “I know now that if something is important to me, I need to do it.”
Total	157		

## 4 Discussion

The present study builds on previous established knowledge and takes a step further to suggest a holistic and integrated approach to increasing well-being in everyday life, as measured by life satisfaction scores. Overall, the findings indicate that the *Activating Values Intervention*—a short-term intervention involving the identification of a valued life domain, selection of an associated activity, and subsequent action—was beneficial for well-being, not only immediately after the intervention but also two weeks later. Furthermore, when compared to a control condition of reflecting on a valued life domain, the findings shed light on an important distinction between reflection and action, suggesting that merely reflecting about or affirming the importance of a valued life domain is not enough, and it may even hinder the ability to achieve or maintain well-being. It was only the combination of awareness, affirmation, and translation into action that led to beneficial outcomes. This aligns with previous evidence suggesting that maladaptive self-reflection might lead to ruminative thoughts that may even lead to depression (Takano and Tanno, 2009; Olatunji et al., 2013), as well as more PTSD symptoms and poorer well-being following a crisis (e.g., Morris and Shakespeare-Finch, 2011; Liu et al., 2021).

More specifically, our findings suggest that an alignment between one’s identified valued life domain and chosen actions may contribute to an increased sense of self-insight, which reflects purposeful striving involved with increased self-knowledge (Wilson and Dunn, 2004), along with the clarity of one’s understanding of one’s thoughts, feelings, and behavior (Grant et al., 2002), enabling the monitoring of cognitive, affective, and behavioral functions of performance (Boyatzis and Howard, 2013). In other words, given that the intervention facilitated increased reflection on one’s perceived valued life domains and chosen actions, it contributed to increased sense of self-insight. Interestingly, the significant contribution to self-insight persists beyond the immediate effects of the intervention, suggesting its potential role in expanding individuals’ awareness. Aligning one’s identified valued life domains and chosen actions may contribute to an increased sense of self-insight is supported by theoretical frameworks as well as empirical research. For example, according to Self-Determination Theory (SDT), acting in accordance with one’s values satisfies the basic psychological need for autonomy, which is essential for self-insight and self-awareness (Ryan and Deci, 2017). In a similar vein, according to the self-concordance model, by aligning actions with valued life domains, individuals can gain self-knowledge and a deeper understanding of their true selves and what truly matters to them (e.g., Sheldon, 2014). Previous empirical studies also suggest that by aligning actions with valued life domains, individuals may experience a sense of authenticity, which can foster self-insight (Sedikides et al., 2017). Furthermore, when individuals act in ways that affirm their valued life domains, they may be more receptive to self-insight and self-reflection, as their sense of self-integrity is reinforced (Critcher and Dunning, 2015). Overall, by acting in ways that are congruent with one’s deeply held values, individuals may experience greater self-awareness, self-knowledge, and a deeper understanding of their true selves. Such self-affirming loop may be created, whereby implementing one’s values solidifies them further. This direction could be further explored in future studies.

Along these lines, the alignment between one’s identified valued life domain and chosen actions was also found to contribute to SOC, which reflects a resource that plays a crucial role in enabling individuals to perceive the world as structured and comprehensible (Antonovsky, 1979, 1987) and encapsulates not only the drive but also the internal and external reservoirs that individuals can tap into when confronting life’s various challenges (Eriksson and Lindström, 2005). However, interestingly, when exploring the ingredients of SOC, we found that while the intervention contributed to the participants’ comprehensibility (the belief that situations are understandable) and manageability (feeling equipped to cope with life’s challenges and demands), it did not affect their meaningfulness (feeling that life makes sense and challenges are worthwhile). This may suggest that when one’s actions are aligned with their deeply held values, it provides a more coherent framework for understanding the world, giving context to the connection between their values and how they navigate various situations. It may also provide a sense of agency—being equipped to manage life’s challenges and demands effectively. Yet, given that meaningfulness is more closely tied to a person’s overall sense of purpose and the intrinsic value they find in their experiences, it may take more time and effort to develop. In other words, while aligning values with actions can positively contribute to comprehensibility and manageability by providing clarity and a sense of control, it may not inherently change one’s fundamental sense of motivation and beliefs about the worthiness of challenges, as these aspects are often deeply rooted in one’s personal worldview. Future studies may further explore this direction, considering the duration and frequency of the intervention, as well as individual differences.

Furthermore, while we did not find a significant treatment-by-time interaction effect for prioritizing meaning, we did find that the intervention significantly contributed to the participants’ prioritization of meaning in their lives. This further corroborates the findings regarding self-insight and SOC, which together may construct a sense of understanding one’s self and the world that supports a self-reinforcing cycle of actively seeking situations and prioritizing activities that are conducive to experiencing meaning in life. In other words, the intervention may reflect a process of self-reflection to make choices that are in harmony with one’s authentic self. Thus, self-awareness plays a crucial role in identifying personal values, aligning daily pursuits accordingly, and refining these selections by recognizing potential shifts in meaning. Such intervention may empower individuals to craft and nurture a sense of personal meaning that is genuine, intimately pertinent, and grounded in everyday experiences.

The findings also point to an interesting direction for future exploration of the different varieties of meaning, corresponding to the need to distinguish between distinct types of meaning: (1) “contemplating meaning”—a more abstract sense of having an overall meaning in life, which includes reflecting and identifying personal values and sources of meaning, represented in our study by the meaningfulness subscale of coherence (Antonovsky, 1987, 1993); and (2) “activating meaning”—a more concrete, experiential presence of meaning in daily life. The lived experience of meaning differs from having an overall sense of meaning. Prioritizing meaning is more oriented toward experiencing and embodying meaning in action. Thus, it may be important to further elucidate meaning in life constructs, including various aspects of cognition (comprehension; “having a sense of meaning in life”), experience (“experiencing meaning in life”), and behavior (prioritizing meaning in daily choices and activities) (see also Russo-Netzer, 2019). This is aligned with recent conceptualizations of meaning as a multifaceted and complex experience, which is sensitive to potential changes in timeframes, circumstances and situations (King and Hicks, 2021; Zambelli and Tagliabue, 2024) as well as socio-cultural context (Chao and Kesebir, 2013), and comprised of various dimensions and facets (George and Park, 2016; Martela and Steger, 2016; Russo-Netzer and Vos, 2024).

Additionally, the intervention was found to significantly decrease levels of psychopathology, defined by symptoms of anxiety, depression, and stress (Lovibond and Lovibond, 1995), even two weeks after the intervention. This may suggest that when individuals are active, in concordance with their values, it contributes to their sense of competence, which may serve as a protective resource that enables better self-regulation in dealing with the increasingly rising stressors and challenges of the modern world. Along the same lines, the explicit articulation of life values and the emphasis on taking committed actions, potentially fostering hope and facilitating behavioral changes, have also been underscored by meta-analyses of interventions based on the ACT model, which demonstrated effects on anxiety and depression (Hofmann et al., 2010; Veehof et al., 2016).

The decreased psychopathology levels may also suggest that the intervention enabled strengthening of participants’ resilience. Taking values-congruent action may have boosted self-efficacy and coping skills, equipping participants to better manage stressors (Southwick et al., 2016). Previous studies also support the contribution of aligning behaviors with personal values to increased life satisfaction and meaning, which may mitigate symptoms of depression and anxiety (Wilson et al., 2010). Focusing on personal values may provide a sense of direction, control and motivation, which counters the hopelessness associated with mental health issues such as depression and anxiety. In general, it may demonstrate the potential contribution of short-term interventions in supporting and providing prevention of psychopathology among nonclinical samples in the community, which are highly needed as complementary to psychotherapy. This promising direction should be explored in greater depth in future studies.

The study also highlights initial insights based on qualitative analyses of the participants’ responses to open-ended questions, which enabled us to develop a deeper understanding of the types and frequency of activities chosen (those emphasizing family relationships were chosen the most, followed by physical activity). It also enabled us to understand what helped participants to attain desired goals in valued life domains—with planning and reminders most cited, followed by a supportive environment—as well as central insights following the intervention, with awareness and self-development and self-efficacy as the main takeaways from the experience. Another insight we derived from the participants’ reports was their emphasis on the importance of investing time and efforts in the process of identifying a valued life area and acting upon it in daily life. These directions suggest new horizons for future studies and emphasize the importance of a mixed methods research approach.

Overall, in accordance with an existing large body of research demonstrating the power of brief situational interventions in promoting purposeful change (see Cohen et al., 2017; Walton and Wilson, 2018) and enduring improvements in life satisfaction (Lyubomirsky and Layous, 2013; Margolis and Lyubomirsky, 2020; Ko et al., 2021), the present study offers a short-term intervention that can be implemented in everyday settings and that appears to be efficient in contributing to individuals’ well-being. Taken together, it is suggested that the combination of awareness, affirmation, and translating values into action contributed to the observed beneficial outcomes. These ingredients reflect the potential underlying psychological processes that contributed to the effectiveness of intervention, by adopting a framework which integrates both flexibility (i.e., allowing participants to choose a valued life domain and take responsibility for corresponding activities that suit them best in terms of type and timing) and structure (requiring participants to commit to selecting and engaging in activities, and providing mechanisms for reporting their experiences). Previous studies on PPIs indicate that individuals who voluntarily engage in such interventions tend to experience greater improvements in life satisfaction than those who do not (e.g., Sin and Lyubomirsky, 2009; Russo-Netzer and Cohen, 2022).

### 4.1 Limitations and suggestions for future research

The present study has several main limitations that should be taken into consideration. First, all outcome measures were assessed simultaneously across all time points, highlighting the need for longitudinal study designs to examine potential causal relationships among them. Future studies should thus adopt a longitudinal approach to explore and refine a more nuanced understanding of the underlying mechanisms of the interplay found between the intervention and each of the various outcomes explored in this study at T2, T3 and T4. More specifically, given that this is a one-time intervention, the observed significant effect persisting for two weeks post-intervention appears reasonable, aligning with prior findings in the literature on Positive Psychological Interventions (PPIs) regarding the optimal duration and frequency of implementing specific interventions. However, it remains unclear what the optimal rate of engagement is for each activity and whether there exists a saturation point where effectiveness diminishes due to factors such as hedonic adaptation. For example, research by Sheldon and Lyubomirsky (2004) revealed that individuals who practiced gratitude exercises once a week experienced greater improvement in well-being compared to those who did so three times a week. This illustrates that the correlation between PPI frequency and well-being is not always straightforward. Consequently, it becomes imperative to diversify PPIs into multiple variations to prevent boredom and adaptation. In the context of the present study, future research endeavors could delve deeper into exploring a range of prospective follow-up interventions, taking into account not only the frequency of engaging in the Activating-Values-Intervention but also considering factors such as response format, delivery mode, and the breadth and depth of content coverage, which may potentially influence the effectiveness of the intervention for specific individuals. Second, given that the participants were all from a Western, moderately individualized culture, it would be worthwhile to examine the generalizability of our findings to samples from various cultures.

Furthermore, although the intervention focused on valued life domains, it did not refer explicitly to values. Future studies may inquire more deliberately about the role of values in the intervention. Another potential limitation arises from the reliance on self-report measures for all outcomes. While suitable for capturing internal motivations and lived experiences (e.g., Sheldon and Lyubomirsky, 2007), this method introduces possible response biases. Subsequent research could mitigate this issue through behavioral assessments or supplementary evaluations from close social connections. Gathering multiperspective data from significant others may reveal observable changes overlooked or misconstrued by participants themselves. Triangulating subjective self-reports with empirical observations could enrich interpretations and validate any self-reported transformations inspired by the intervention.

Overall, the present study furnishes initial evidence that purposeful engagement in value-aligned behaviors may cultivate well-being. It offers a useful contribution by providing a concrete protocol that enables individuals to plan and intentionally undertake action in a valued life domain. Integrating such interventions in a routine manner in daily life may open new directions for implementing positive change, personal development, and better mental health. Previous studies similarly show that brief, scalable interventions can support primary prevention goals when applied to promote well-being and resilience across nonclinical community samples (e.g., Bolier et al., 2013; Russo-Netzer and Cohen, 2022). As such, the findings hold meaningful implications for therapeutic, organizational, and educational initiatives seeking to empower individuals’ flourishing.

## Data availability statement

The raw data supporting the conclusions of this article will be made available by the authors, without undue reservation.

## Ethics statement

The studies involving humans were approved by Achva Academic College Approval No. 0182. The studies were conducted in accordance with the local legislation and institutional requirements. The participants provided their written informed consent to participate in this study.

## Author contributions

PR-N: Conceptualization, Writing – original draft, Writing – review & editing. OIA: Conceptualization, Methodology, Writing – review & editing.
